# Activation of TP53 target genes in the primary response of triple-negative breast cancer cells to doxorubicin treatment

**DOI:** 10.1038/s41598-025-31087-x

**Published:** 2025-12-04

**Authors:** Aysan Shekari, Yaghub Pazhang, Hamid Maadi

**Affiliations:** 1 Department of Biology, Faculty of Sciences, Urmia University, Urmia, Iran; 2 Department of Cellular and Molecular Biotechnology, Institute of Biotechnology, Urmia University, Urmia, Iran; 3 Department of Cell and Molecular Biology & Microbiology, Faculty of Biological Science and Technology, University of Isfahan, Isfahan, Iran

**Keywords:** Triple-negative breast cancer, Doxorubicin, DNA damage, TP53 target genes, Breast cancer, Cancer therapy

## Abstract

Among the DNA-damaging agents commonly used in clinical settings doxorubicin has emerged as one of the most effective treatments for Triple-negative breast cancer (TNBC). Our limited understanding about the molecular mechanisms underlying the short- and long-term responses of TNBC cells to DNA damage induced by drugs like doxorubicin is a hurdle to improve the efficacy of the treatment or overcome the drug resistance. In this study, we aimed to elucidate the immediate response of the TNBC cells to doxorubicin and compare these responses with those of doxorubicin-resistant cells through transcriptome analysis. Transcriptome analysis revealed that doxorubicin significantly upregulates the expression of TP53 target genes, including *CDKN1A*, *TIGAR*, *TP53INP1*, *PPM1D*, and *ACER2*. Notably, doxorubicin-resistant TNBC cells failed to increase the expression of these genes, except for *CDKN1A*, upon doxorubicin treatment. Moreover, treatment with etoposide as another DNA-damaging drug increased the expression of *CDKN1A*, *TP53INP1*, and *ACER2* in a TP53-independent manner. Collectively, this study highlights the critical role of TP53 target genes in the immediate response of TNBC cells to DNA-damaging agents like doxorubicin and etoposide. It also reveals distinct molecular mechanisms regulating their expression in resistant versus sensitive cells, offering potential therapeutic targets to improve treatment strategies for TNBC.

## Introduction

 Triple-negative breast cancer (TNBC) is characterized by the absence of estrogen receptor (ER), progesterone receptor (PR), and human epidermal growth factor receptor 2 (ErbB2) expression^1,2^. It accounts for 10–20% of all patients diagnosed with breast cancer (BC) and is associated with a high frequency of metastasis and poor prognosis^3,4^. Several therapeutic strategies have been proposed for TNBC treatment, with conventional systemic chemotherapy becomes the mainstay for the treatment of these patients^5,6^.

Doxorubicin is a widely recognized anticancer drug that induces DNA damage by inhibiting the normal function of topoisomerase II. It intercalates between DNA strands, leading to DNA double-strand breaks (DDBs)^6–8^. This drug is commonly used to treat various types of cancers, including BC, lung cancer, and several types of blood cancers^9–11^. Among anthracyclines, doxorubicin is one of the most effective agents and is frequently used to treat patients with TNBC^12,13^. However, despite its ability to improve survival rates in TNBC patients, some individuals do not respond or develop resistance to the treatment.

One of the major challenges in distinguishing between responders and non-responders to doxorubicin treatment is the limited understanding of the molecular mechanisms underlying the short-term response of TNBC cells to the drug and its association with drug resistance. The immediate cellular response to DNA damage can critically influence cell fate, either enabling effective repair of DNA DDBs or triggering cell cycle arrest and apoptosis as a result of extensive DNA damage accumulation. Therefore, understanding the mechanisms behind these divergent responses in TNBC cells is critical for improving therapeutic outcomes.

In this study, we employed RNA-seq and microarray datasets to investigate the short-term effects of doxorubicin as a DNA-damaging drug on gene expression profile and signaling pathways activated in TNBC cells. Transcriptome analysis of CAL51 and HCC1806 TNBC cell lines treated with doxorubicin revealed seven differentially expressed genes (DEGs) common to both cell lines. Interestingly, all upregulated genes, including *CDKN1A*, *TIGAR*, *TP53INP1*, *PPM1D*, and *ACER2* were identified as tumor protein 53 (TP53) direct targets. Consistent with these findings, quantitative PCR (qPCR) analysis demonstrated that doxorubicin elevates the expression of *CDKN1A*, *TP53INP1*, and *PPM1D* in MDA-MB-231 cells. However, in doxorubicin-resistant CAL51 cells, only *CDKN1A* was significantly upregulated upon doxorubicin treatment, based on a predefined threshold. Furthermore, treatment of MDA-MB-231 cells with etoposide, another DNA-damaging agent, resulted in increased expression of *CDKN1A*, *TP53INP1*, and *ACER2*, independent of TP53 function.

## Materials and methods

### Cell line and treatment

The MDA-MB-231 cell line was purchased from the Iranian Pasteur Institute Cell Bank. Cells were cultured in RPMI 1640 medium (Gibco, USA) supplemented with 10% fetal bovine serum (FBS, Gibco), 1% penicillin-streptomycin (100X, Sigma), and L-glutamine. Cultures were maintained at 37 °C with 95% humidity and 5% CO2. Cells were routinely passaged at 80% confluency at a ratio of 1:3. The culture medium was changed every 72 h. Working cell banks were created from second-passage cultures, and all experimental assays were performed on the first passage after thawing these vials.

Doxorubicin was purchased from Sigma and dissolved in distilled water. A range of doxorubicin concentrations (2.5, 5, 10, 20, and 40 µg/mL) was prepared for treating the cancer cells.

### MTT assay

To assess cell viability, 5000 cells were seeded per well in a 96-well plate and treated with varying concentrations of doxorubicin for 24 h. After incubation, 10 µL of MTT solution (3-(4,5-dimethylthiazol-2-yl)−2,5-diphenyltetrazolium bromide; 5 mg/mL, Sigma) was added to each well, and the plate was incubated at 37 °C for 3–4 h. To solubilize the formazan crystals, 100 µl of dimethyl sulfoxide (DMSO, Merck) was added to each well. The plate was then gently shaken on a low-speed shaker for 15 min. Finally, the absorbance was measured at 570 nm using a Biotek ELISA reader, and cell viability was calculated accordingly^14^.

### Real-time PCR

The half-maximal inhibitory concentration (IC50) of doxorubicin was determined using Prism software version 10.3.1 (GraphPad Software, La Jolla, CA, USA). The IC50 value was calculated based on cell viability results obtained after treating the cells with a range of doxorubicin concentrations for 24 h.

For RNA extraction, cells were treated with 20.87 µg/mL of doxorubicin (the calculated IC50 value) for 24 h. Total RNA was then extracted using the RNXplus kit (Sinaclon, Iran) according to the manufacturer’s instructions. The concentration and purity of the extracted RNA were measured using a Nanodrop spectrophotometer (Biotek).

Next, cDNA was synthesized from the RNA using a cDNA synthesis kit (Parstus, Iran) according to the manufacturer’s protocol. Normalized concentrations of cDNA, along with primers specific to *TIGAR*, *CDKN1A*, *TP53INP1*, *PPM1D*, and *Beta-Actin*, were used for real-time PCR (Applied Biosystems). All primers were designed using Oligo 7 software, and their sequences are provided in Table 1.

**Table 1:** The primer sequences used for qPCR.

Gene	Primer	Primer sequence 5’−3’
***TP53INP1***	Forward	GAACATCCCAGCATGTCTGTC
	Reverse	GAGCTTCCACTCTGGGACTAC
***TIGAR***	Forward	CGGGGTTGTAGAAGGCAAAG
	Reverse	TCCACGCATTTTCACCTGGT
***CDKN1A***	Forward	ACCTGTCACTGTCTTGTACCC
	Reverse	ATCTGTCATGCTGGTCTGCC
***PPM1D***	Forward	TGTGGAAGAAACTGGCGGAA
	Reverse	ACATACATCTTCATGCCCCGA
***β-Actin***	Forward	CAACTGGGACGACATGGAGAAA
	Reverse	GATAGCACAGCCTGGATAGCAA

### RNA-seq data analysis

All in silico analyses were performed using R version 4.4.2 and RStudio version 2023.06.0.421. Relevant RNA-seq datasets were selected from the Gene Expression Omnibus (GEO) database, and raw sequencing data with accession numbers of GSE174692 ^15^ and GSE181010 ^16^ were downloaded from the Sequence Read Archive (SRA) using the SRA toolkit. Detailed information about each dataset is summarized in Table 2.

The raw RNA-seq reads were aligned to a reference transcriptome index generated from reference transcriptome (Ensembl; *Homo sapiens* version 86) Using Kallisto version 0.46.1^17^. Transcript-level abundances were summarized to gene-level counts using the tximport package^18^. The resulting gene-level counts were filtered and normalized using the trimmed mean of M values (TMM) method from the edgeR package version 3.42.4^19^.

To assess sample clustering and identify potential outliers, Principal Component Analysis (PCA) was performed on the normalized data^20^. Differential expression analysis was conducted using the limma package version 3.56.2^21^. The voom function was applied to estimate the mean-variance relationship, followed by linear modeling to identify DEGs. Genes with a False Discovery Rate (FDR) ≤ 0.05 and an absolute log2 Fold Change (logFC) ≥ 1 were considered statistically significant DEGs.

The DEGs were visualized by heatmap and volcano plots using gplots (version 3.1.3.1)^22^ and ggplot2 (version 3.4.4)^23^ packages, respectively. The venn diagram were generated using VennDiagram package version 1.7.3^24^.

**Table 2:** Detailed information about each dataset.

Accession	Organism	Samples	Platform	Reference
GSE174692	*Homo sapiens*	GSM5322577, GSM5322578, GSM5322579, GSM5322580, GSM5322581, GSM5322582	Illumina NovaSeq 6000	^13^
GSE181010	*Homo sapiens*	GSM5481983, GSM5481984, GSM5481985, GSM5481986, GSM5481987, GSM5481988, GSM5481989, GSM5481990, GSM5481991, GSM5481992, GSM5481993, GSM5481994	Illumina NextSeq 500	^14^
GSE202536	*Homo sapiens*	GSM6123775, GSM6123776, GSM6123777, GSM6123778, GSM6123779, GSM6123780, GSM6123784, GSM6123785, GSM6123786, GSM6123787, GSM6123788, GSM6123789	Affymetrix Human Genome U133 Plus 2.0 Array	^23^

### Microarray data analysis

Raw microarray data with the accession number GSE202536 were downloaded from GEO database^25^. The raw data were processed and normalized using ReadAffy and rma functions from the affy package version 1.78.2^26^. PCA analysis was performed as described in the previous section. Finally, linear modeling was applied to identify DEGs using the limma package.

### Drug sensitivity analysis

The IC50 (µM) values for doxorubicin, epirubicin, etoposide, and mitoxantrone were extracted from the Genomics of Drug Sensitivity in Cancer (GDSC) database^27^ (Release 8.5). These values, representing drug sensitivity across various TNBC cell lines, were visualized and analyzed using Prism software version 10.3.1 (GraphPad Software, La Jolla, CA, USA).

### Genetic alterations and expression of DEGs in BC

To evaluate genetic alterations of genes in BC, both BC cell lines and breast tumor samples were analyzed using data from the cBioPortal cancer genomics database^28–30^. For cell line analysis, genetic alteration data were extracted from the Cancer Cell Line Encyclopedia, Broad 2019 dataset (1739 samples)^31,32^. For tumor samples the following datasets were utilized: METABRIC Nature 2012 & Nat Commun 2016 (2509 samples)^33–35^, MSK Cancer Cell 2018 (1918 samples)^36^, SMC 2018 (187 samples)^37^, TCGA Firehose Legacy (1108 samples), INSERM PLoS Med 2016 (216 samples)^38^, Archived 2020 (237 samples), and Provisional, December 2021 (379 samples). The data were visualized using Prism software version 10.3.1 (GraphPad Software, La Jolla, CA, USA).

To assess gene expression levels across different types of BC, the GEPIA 2 database^39^ was used. GEPIA 2 integrates original data from The Cancer Genome Atlas Program (TCGA) and the Genotype-Tissue Expression (GTEx) databases.

### Protein-protein interaction network and functional enrichment analysis

The STRING database version 12.0^40^ was used to construct a protein-protein interaction (PPI) network, identifying both direct and indirect interactions among the proteins of interest. In addition, Gene Ontology (GO) enrichment analysis was performed using the STRING database to functionally annotate the selected genes and explore their biological roles.

### DepMap database analysis

Gene dependency data were obtained from the DepMap (Dependency Map) public database (DepMap Public 25Q2, Broad Institute)^41^. TNBC cell lines were selected form database. Gene effect scores were derived from genome-wide CRISPR-Cas9 loss-of-function screens using the Chronos model, where negative values indicate higher gene dependency. Drug sensitivity profiles were obtained from the GDSC dataset, represented as log-transformed IC50 (µM).

Correlation analyses were carried out to evaluate associations between gene dependency scores and drug sensitivity across TNBC cell lines. Data were plotted to visualize these relationships, with regression lines fitted to show the linear trends.

### Statistical analysis

Data were analyzed using one-way analysis of variance (ANOVA) followed by the Tukey post hoc test in Prism software version 10.3.1 (GraphPad Software, La Jolla, CA, USA). Results are presented as mean ± standard deviation, and a p-value < 0.05 was considered statistically significant.

## Results

### Sensitivity of TNBC cell lines to DNA-damaging drugs

To evaluate the sensitivity of TNBC cell lines to DNA-damaging drugs, the IC50 (µM) values for doxorubicin, epirubicin, etoposide, and mitoxantrone were obtained from GDSC database. These drugs are known to induce DNA double-strand breaks (DSBs) by inhibiting the normal function of topoisomerase II^42,43^.

**Fig. 1:**
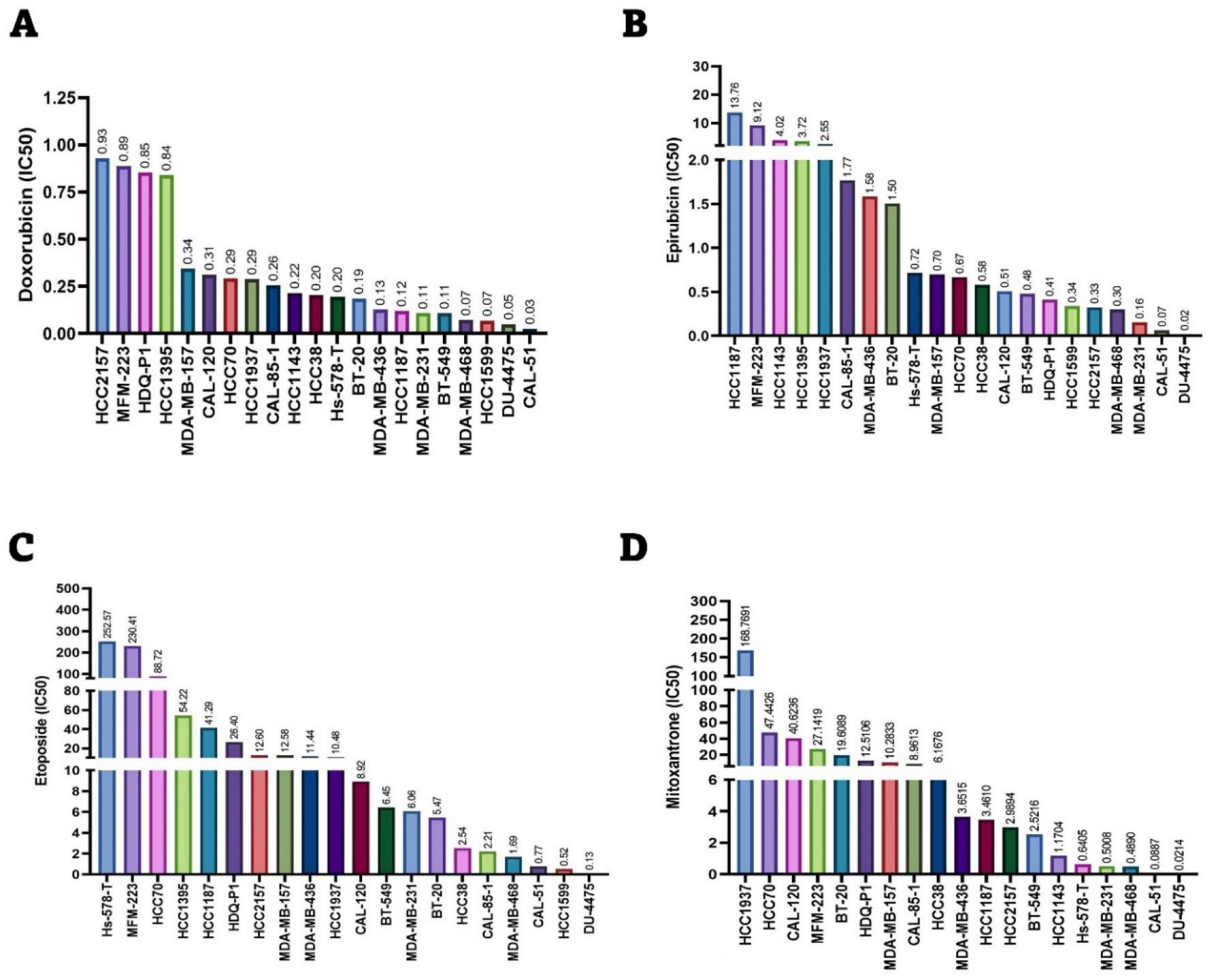
Sensitivity of TNBC cell lines to various DNA-damaging drugs. The IC50 values for (**A**) doxorubicin, (**B**) epirubicin, (**C**) etoposide, and (**D**) mitoxantrone were obtained from GDSC database. The IC50 value for each drug in each cell line is indicated above the corresponding bar in the chart. All IC50 values are expressed in micromolar (µM).

As shown in Fig. 1, the sensitivity of TNBC cell lines varies significantly depending on the drug. For instance, some TNBC cell lines, such as MFM-223, exhibit higher resistance to DNA-damaging drugs, while others, like CAL51 and MDA-MB-231 are more sensitive. The observed differences in drug sensitivity among TNBC cell lines may reflect variations in DNA repair mechanisms, drug uptake, or other cellular adaptations. It is worth noting that, according to the TP53 Database^44^, among the three cell lines used in this study, CAL51 harbors a WT *TP53* gene, whereas HCC1806 and MDA-MB-231 carry loss-of-function mutations.

### Gene expression patterns of TNBC cells treated with doxorubicin

To evaluate the short-term effects of doxorubicin on gene expression and cellular signaling in TNBC cells, raw RNA-seq (GSE174692) and microarray (GSE202536) data were obtained from GEO database.

In the GSE174692 datasets, HCC1806 (a human TNBC cell line) was treated with either DMSO (control) or 1 µM doxorubicin for 24 h. In the microarray dataset (GSE202536), CAL51 TNBC cells were treated with 0.4 µM doxorubicin for 24 and 48 h. For consistency with the RNA-seq dataset, the 24-hour treatment data were selected for analysis.

**Fig. 2:**
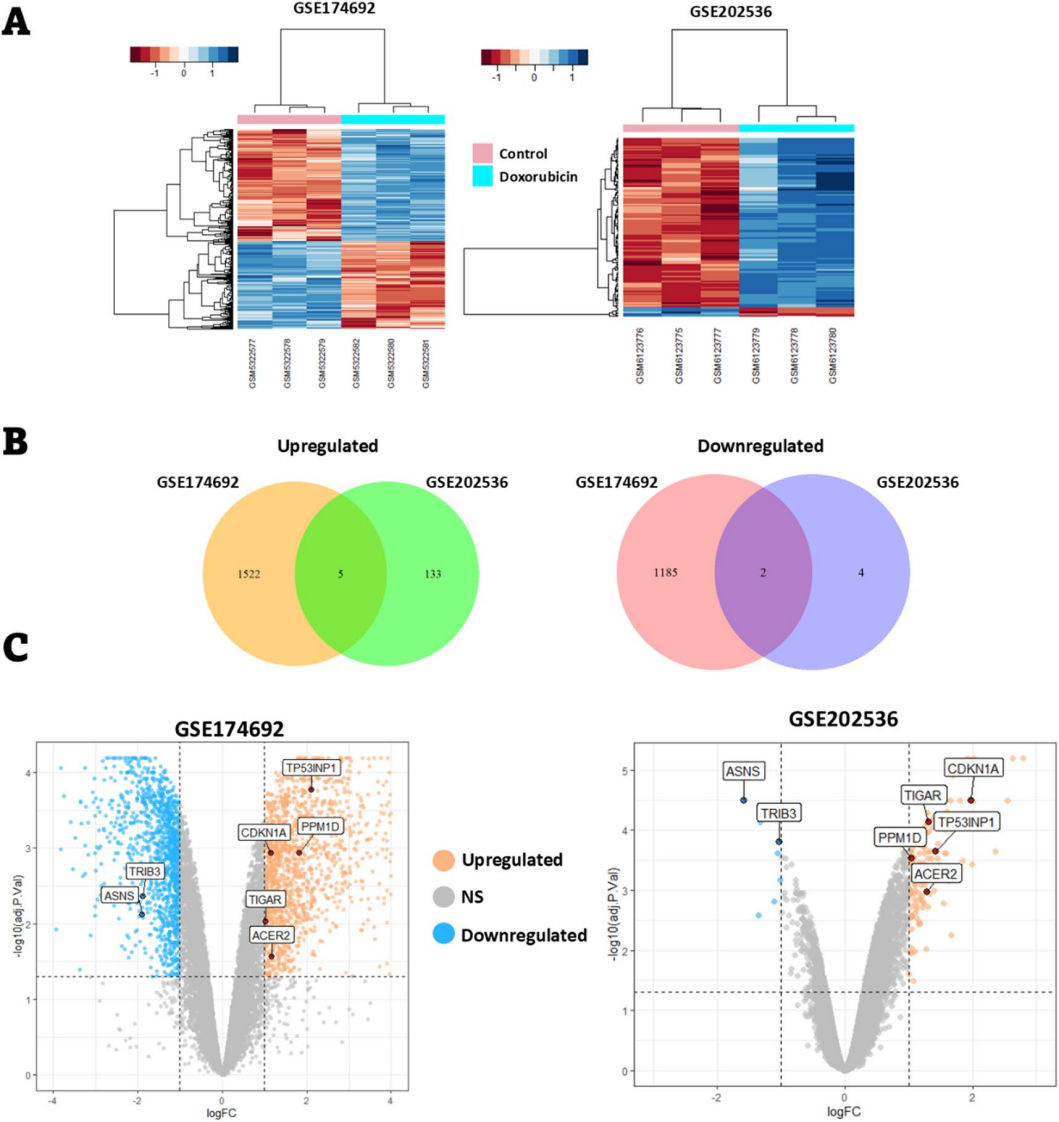
Differential expression analysis of genes in TNBC cell lines treated with doxorubicin. (**A**) The heatmap illustrates DEGs in HCC1806 (GSE174692) and CAL51 (GSE202536) cells treated with 1 µM and 0.4 µM doxorubicin, respectively, for 24 h. Each column represents a sample, and each row represents a DEG. Three replicates for both control and doxorubicin-treated groups are shown, labeled with their respective sample accessions numbers (GSM). DEGs were defined as genes with a logFC ≥ 1 and adjusted p-value (adj.P.value) ≤ 0.05. (**B**) For the GSE174692 dataset, 1527 genes were upregulated, and 1187 genes were downregulated following doxorubicin treatment. In the GSE202536 dataset, 138 genes were upregulated, and 6 genes were downregulated after treatment of the cells with doxorubicin. Five genes were commonly upregulated, and two genes were commonly downregulated in both datasets. DEGs were defined as genes with a logFC ≥ 1 and adj.P.value ≤ 0.05. (**C**) The volcano plots highlight the common DEGs identified in both datasets. The x-axis represents the logFC, and the y-axis represents the -log10 of the adj.P.value. DEGs were defined as genes with a logFC ≥ 1 and adj.P.value ≤ 0.05. Gene symbols are indicated in the boxes.

Our analysis revealed 1527 and 138 upregulated genes in the GSE174692 and GSE202536 datasets, respectively, following doxorubicin treatment (Fig. 2A and B). Among these, five genes were commonly upregulated in both datasets **(**Fig. 2B**)** including TP53 Induced Glycolysis Regulatory Phosphatase (*TIGAR*), Tumor Protein p53 Inducible Nuclear Protein 1 (*TP53INP1*), Alkaline Ceramidase 2 (*ACER2*), Cyclin Dependent Kinase Inhibitor 1 A (*CDKN1A*), and Protein Phosphatase, Mg^2+^/Mn^2+^ Dependent 1D (*PPM1D*) genes (Fig. 2C). In addition, 1187 and 6 genes were downregulated in the GSE174692 and GSE202536 datasets, respectively (Fig. 2A and B). Two genes were commonly downregulated in both datasets **(**Fig. 2B**)** including Tribbles Pseudokinase 3 (*TRIB3*) and Asparagine Synthetase (Glutamine-Hydrolyzing) (*ASNS*) genes (Fig. 2C). Taken together, analysis of doxorubicin’s effects on gene expression in two different TNBC cell lines across two independent experiments identified seven common DEGs in both datasets.

### Identification of protein-protein interaction networks in common DEGs

**Fig. 3:**
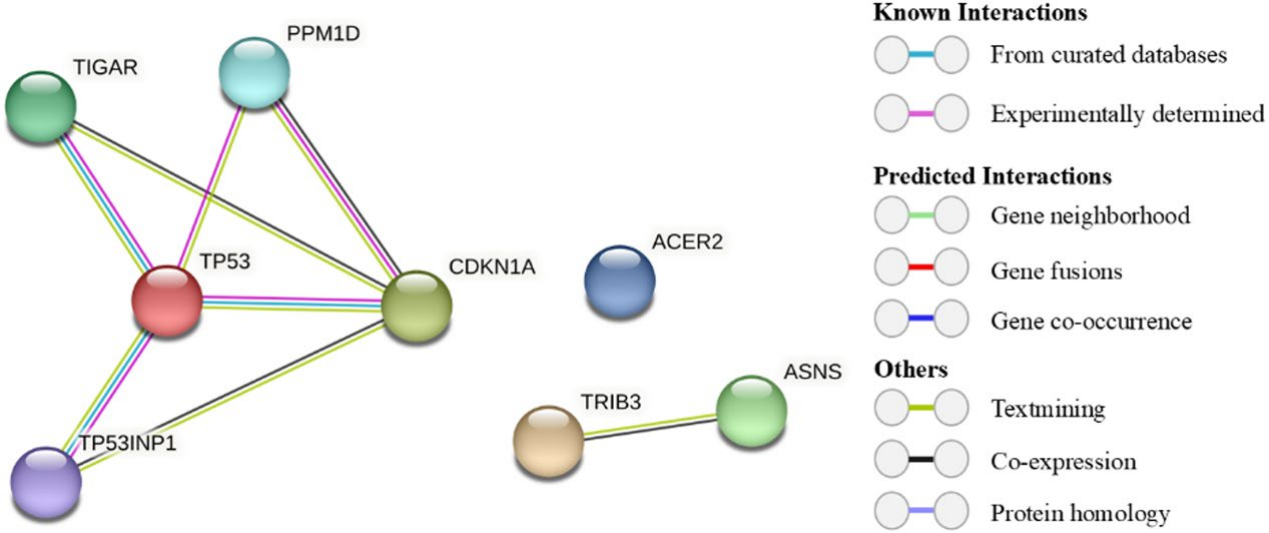
PPI network analysis of common DEGs. Each colored node represents a protein, illustrating its potential interactions with other proteins. The colored lines connecting the nodes indicate the nature of the PPI, with the specific types of interactions corresponding to each color displayed on the left. The network highlights the interaction between TP53 and four commonly upregulated genes.

To explore potential interactions among common DEGs, we constructed a PPI network using the STRING database. The analysis identified two distinct networks derived from upregulated and downregulated genes (Fig. 3). Gene Ontology (GO) analysis indicated that all DEGs are involved in the cellular response to stress, which is consistent with the DNA damage and cellular stress induced by doxorubicin (Table 3).

The common upregulated genes were found to be associated with DNA damage response, response to radiation, and positive regulation of cell death (Table 3), suggesting their activation in response to DNA damage caused by doxorubicin in TNBC cells. Further investigation into the role of these common DEGs in the cellular response to DNA damage stress revealed that all the common upregulated genes are direct targets of TP53^44^. The PPI network analysis demonstrated the experimentally validated interactions between TP53 and four of the common upregulated genes (Fig. 3). Although the PPI network did not show a direct link between ACER2 and TP53, previous studies have established that ACER2 is a direct target of TP53^45^. Taken together, our PPI network analysis highlights the critical role of common DEGs in cellular processes activated during DNA damage stress induced by doxorubicin.

**Table 3:** Functional enrichment analysis using the gene ontology (GO) enrichment analysis.

Biological process		
Description	False discovery rate (FDR)^*^	Matching genes
Cellular response to stress	0.00033	TIGAR, PPM1D, ACER2, TP53INP1, ASNS, CDKN1A, TRIB3
DNA damage response, signal transduction by p53 class mediator	0.0035	PPM1D, ACER2, CDKN1A
Regulation of autophagy	0.0172	TIGAR, ACER2, TP53INP1, TRIB3
Response to radiation	0.0228	TIGAR, PPM1D, TP53INP1, CDKN1A
Cellular response to starvation	0.0437	PPM1D, ASNS, CDKN1A
Positive regulation of cell death	0.0464	TIGAR, ACER2, TP53INP1, CDKN1A

^*^The FDR ≤ 0.05 considered as statistically significant.

### Identification of expression and genetic alterations of common DEGs in various types of BCs

**Fig. 4:**
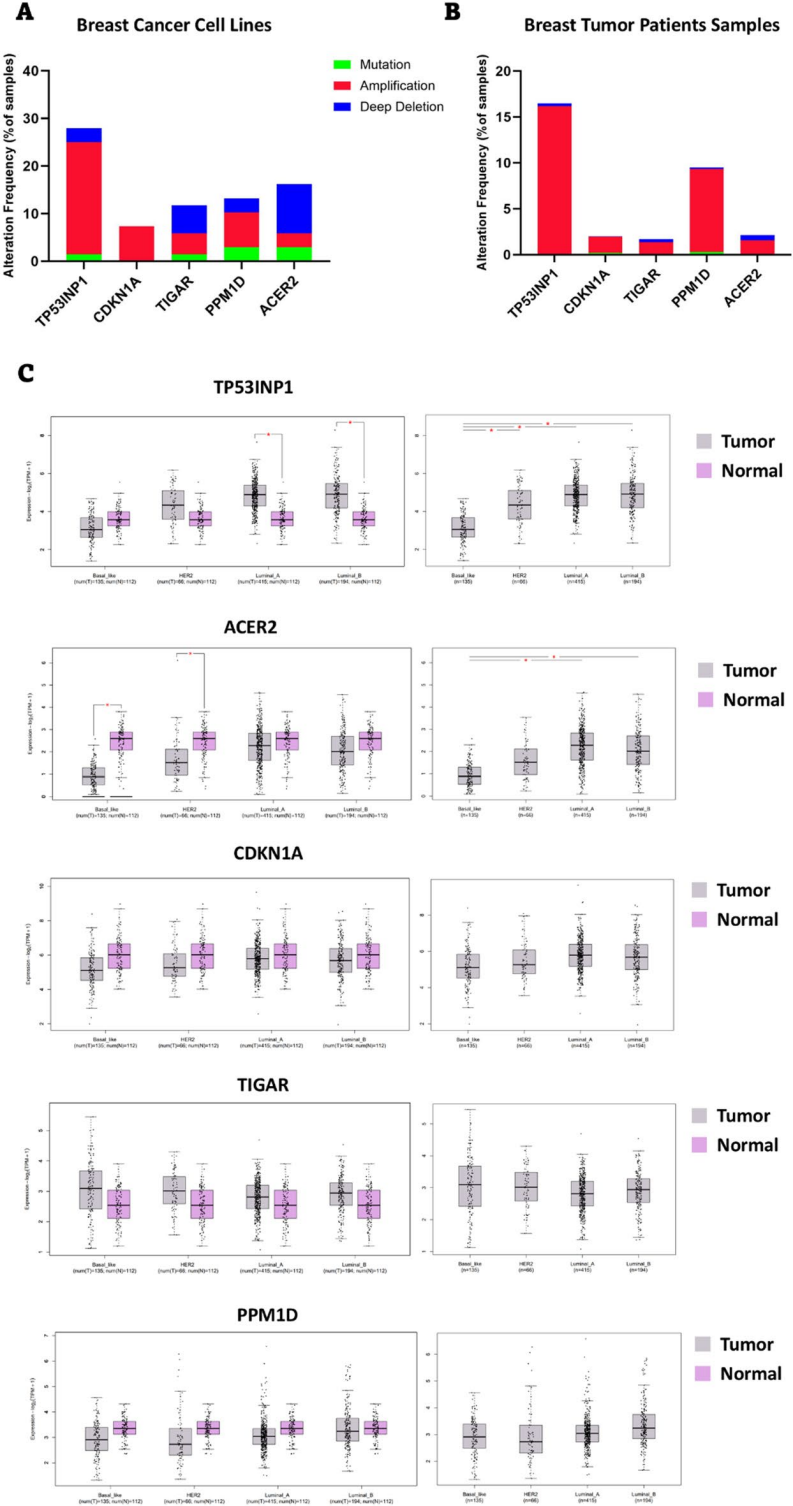
Genetic alterations and expression patterns of common upregulated DEGs in breast tumors. The frequency of genetic sequence alterations and copy number changes in common DEGs was analyzed using genome sequencing data from (**A**) breast cancer cell lines and (**B**) breast tumor samples (6554 samples). Data were obtained from the cBioPortal cancer genomics database and are presented as stacked bar charts. (**C**) The left panel shows the expression levels of common DEGs in normal samples compared to breast tumor samples across different breast cancer subtypes. The right panel compares the expression of these DEGs between different breast cancer subtypes. Data were obtained from the GEPIA 2 database. * *p* < 0.05 indicates statistical significance.

Next, we evaluated the genetic alterations and expression patterns of common DEGs across different types of BC. To better understand the role of TP53 in the response of TNBC cells to DNA-damaging drugs, we focused on TP53 direct target genes (common upregulated DEGs). Using the cBioPortal database, we analyzed the frequency of mutations, amplifications, and deletions in five common upregulated DEGs across various BC cancer cell lines and tumor samples. Our results revealed that the *TP53INP1* gene is amplified in approximately 23% of cancer cell lines (Fig. 4A) and 16% of breast tumors (Fig. 4B). Analysis of *TP53INP1* expression across different cancer types using the GEPIA 2 database^39^ showed that this gene is highly expressed in luminal A and luminal B subtypes of BC (Fig. 4C). Interestingly, *TP53INP1* expression was significantly downregulated in TNBC tumors compared to other BC subtypes (Fig. 4C). In addition, we found that the expression of the *ACER2* gene is significantly downregulated in TNBC and HER2-positive breast tumor samples compared to normal samples (Fig. 4C). Further comparison of *ACER2* expression across various types of breast tumor types revealed lower expression levels in TNBC tumors compared to other subtypes (Fig. 4C). Consistent with these findings, our analysis showed that the *ACER2* gene is deleted in approximately 10% of BC cell lines (Fig. 4A), while the deletion frequency is much lower in BC patient samples (Fig. 4B). For *CDKN1A*, *TIGAR*, and *PPM1D*, although some genetic alterations were observed in BC cell lines (Fig. 4A) and tumor samples (Fig. 4B), no significant changes in gene expression were detected across different BC subtypes (Fig. 4C). These findings highlight distinct patterns of genetic alterations and expression levels of common upregulated DEGs in various BC subtypes, providing insights into their potential roles in BC biology.

### Evaluation of expression of common upregulated DEGs in MDA-MB-231 cells treated with doxorubicin

**Fig. 5:**
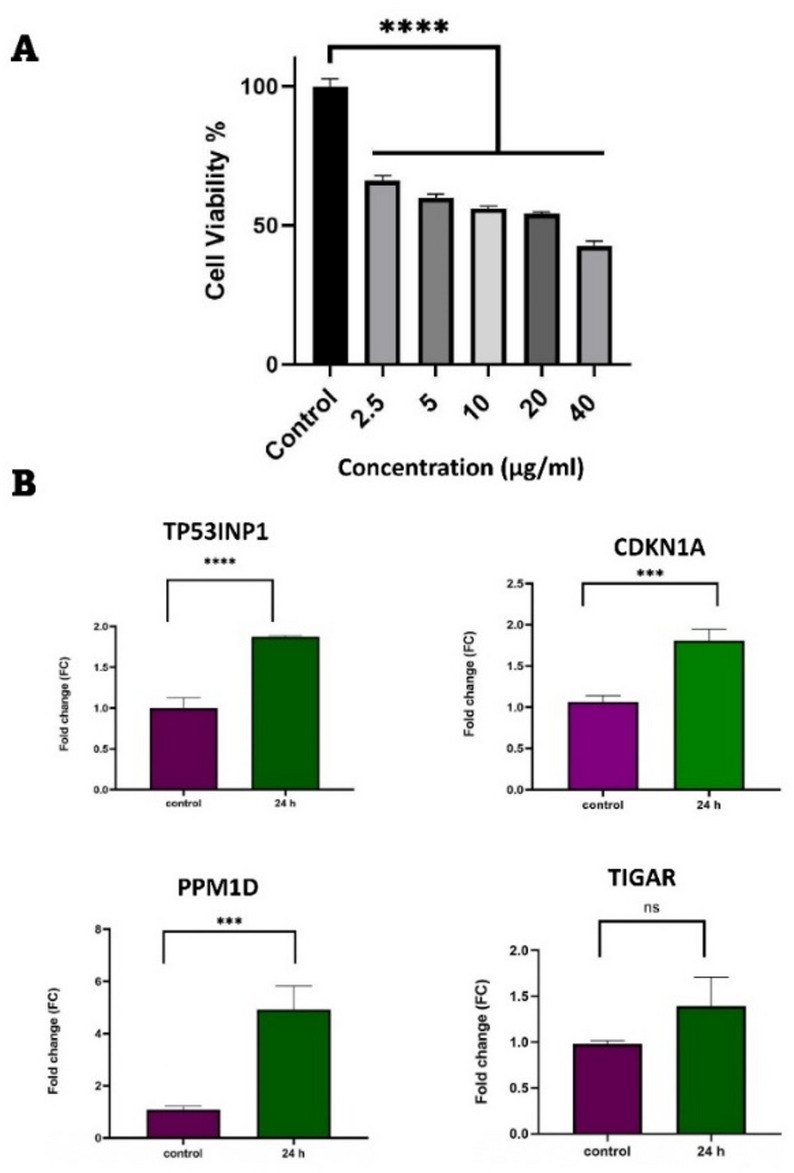
Evaluation of common DEGs expression in MAD-MB-231 TNBC cells treated with doxorubicin. (**A**) MDA-MB-231 cells were treated with 0, 2.5, 5, 10, 20, and 40 µg/mL of doxorubicin, and cell viability was assessed using the MTT assay. Each value represents the average of three experiments, with error bars indicating the standard error. (**B**) Cells were treated with 20.87 µg/mL of doxorubicin for 24 h, and the expression of selected genes was evaluated using qPCR. Statistical significance is indicated as follows: ***: *p* < 0.001, ****: *p* < 0.0001, and ns: not significant.

To determine the appropriate concentration of doxorubicin for gene expression analysis, MDA-MB-231 TNBC cells were treated with doxorubicin at concentrations ranging from 2.5 (4.6 µM) to 40 (73.6 µM) µg/mL, and cell viability was assessed using the MTT assay. The results show that doxorubicin reduces cell viability in a dose-dependent manner (Fig. 5A). The calculated IC50 value, based on cell viability, was 20.87 µg/mL (38.4 µM) of doxorubicin.

We selected *PPM1D*, *CDKN1A*, *TP53INP1*, and *TIGAR*, genes that formed a distinct network in our PPI network analysis, to validate our previous results. In-silico analysis indicated that these genes are upregulated in TNBC cells following 24-hour treatment with doxorubicin (Fig. 2C). To confirm this, MDA-MB-231 TNBC cells were treated with 20.87 µg/mL of doxorubicin for 24 h. Consistent with our in-silico results, qPCR analysis demonstrated that doxorubicin increased the expression levels of *PPM1D* (*p* < 0.001), *CDKN1A* (*p* < 0.001), and *TP53INP1* (*p* < 0.0001) (Fig. 5B). However, no significant change in *TIGAR* expression was observed, which may be explained by the heterogeneity of the TNBC cell lines used in our in silico and in vitro analysis and their variable responses to drug treatment.

These findings validate the upregulation of key genes in response to doxorubicin treatment, supporting their potential role in the cellular response to DNA damage in TNBC cells.

### Evaluation of common DEGs expression in doxorubicin-resistant TNBC cells

**Fig. 6:**
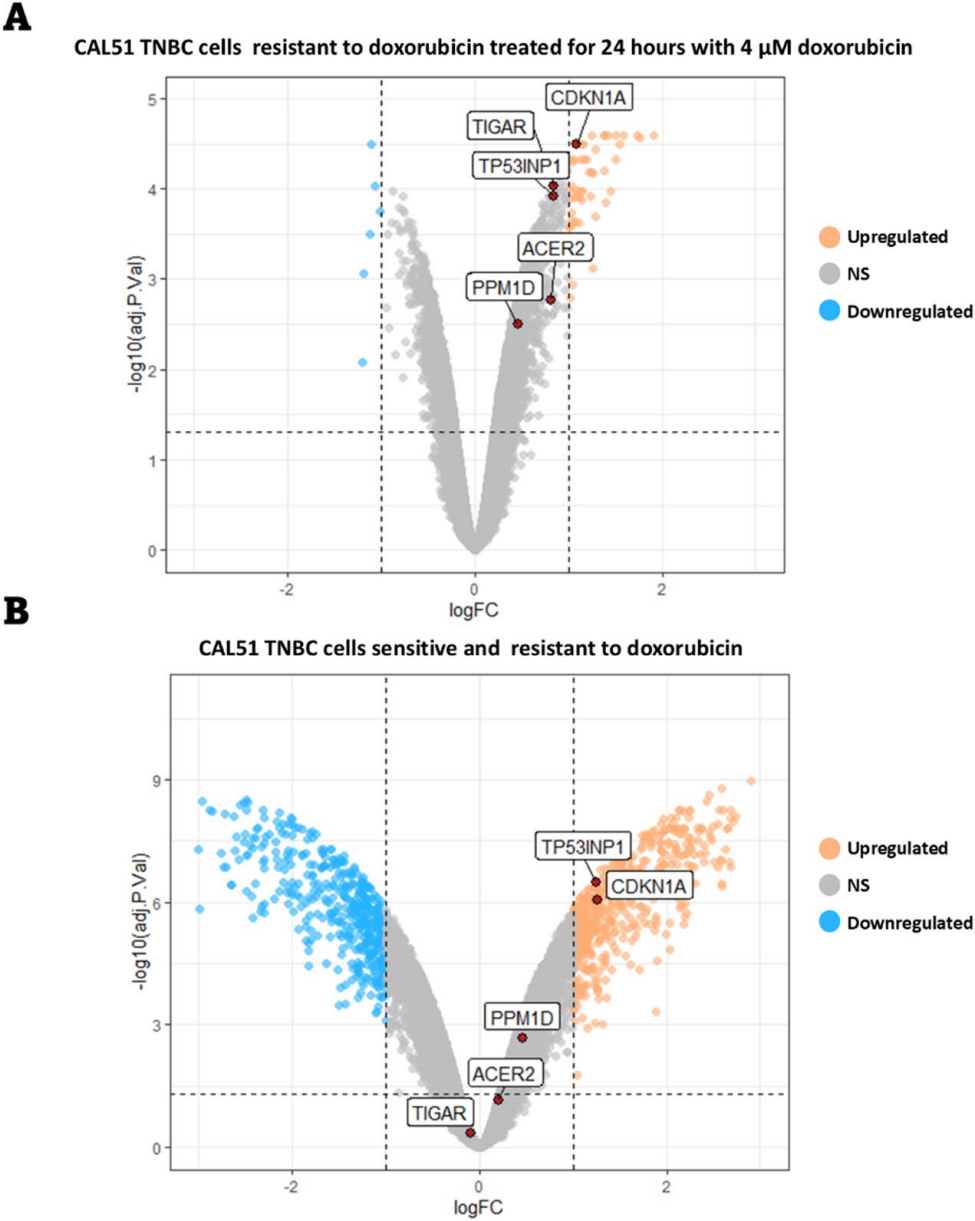
Expression of common DEGs in doxorubicin-resistant CAL51 cells. (**A**) The expressions of common DEGs in doxorubicin-resistant CAL51 cells treated with 4 µM doxorubicin (form the GSE202536 dataset) were compared with untreated control samples. (**B**) Expression levels of common DEGs were compared between doxorubicin-resistant and -sensitive CAL51 cells. The data are presented as a volcano plot, with the x-axis representing the logFC and the y-axis representing the -log 10 of the adj.P.value. Genes with logFC ≥ 1 and adj.P.value ≤ 0.05 were considered as a DEG.

To determine whether doxorubicin resistance of TNBC cells affects their response to the drug, the expression of common DEGs were evaluated in doxorubicin-resistant TNBC cells. For this purpose, we utilized the microarray dataset GSE202536, which includes data from CAL51 TNBC cells that were rendered resistant to doxorubicin and subsequently treated with 4 µM doxorubicin for 24 h. Consistent with previous findings, genes with FDR of less than 0.05 and logFC greater than 1 were identified as significantly upregulated or downregulated genes. Our analysis revealed that only *CDKN1A* was upregulated after treatment of the cells with doxorubicin (Fig. 6A), suggesting that resistant cells suppress the activation of common upregulated DEGs in response to doxorubicin.

To further explore the impact of doxorubicin resistance on the expression of common DEGs, we compared gene expression profiles between doxorubicin-sensitive and -resistant CAL51 cells using the same dataset. The results showed that resistant cells upregulated the expression of *CDKN1A* and *TP53INP1* (Fig. 6B). Collectively, these findings indicate that TNBC cells undergoing doxorubicin resistance alter the signaling pathways involved in the cellular response to DNA damage stress, potentially contributing to their survival under drug treatment.

### Evaluation of common DEGs expression in TNBC cells treated with etoposide

**Fig. 7:**
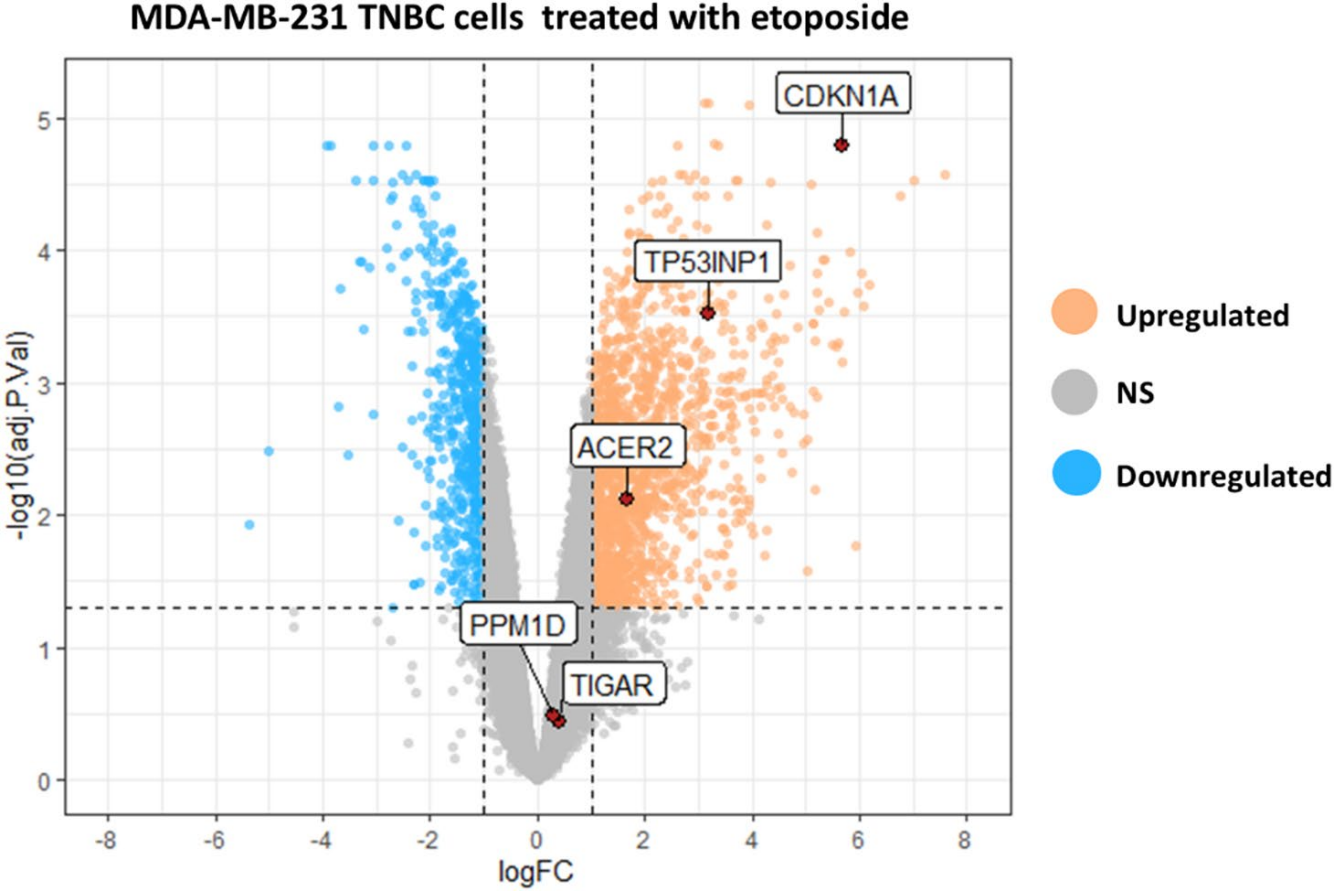
Expression of common DEGs in MAD-MB-231 cells treated with etoposide. The expression of common DEGs in MDA-MB-231 cells treated with etoposide (from RNA-seq dataset GSE181010) were compared with untreated control samples. The data are presented as a volcano plot, with the x-axis representing the logFC and the y-axis representing the -log 10 of the adj.P.value. Genes with logFC ≥ 1 and adj.P.value ≤ 0.05 were considered as a DEG.

Next, we explored the impact of other DNA damaging drugs on the expression of common DEGs in TNBC cells. For this purpose, we used the dataset GSE181010 to examine the effect of etoposide on the gene expression profile of MDA-MB-231 TNBC cells. Etoposide is a DNA-damaging agent that inhibits topoisomerase II function, leading to DNA DSBs^46^. Consistent with our findings for doxorubicin, treating the cells with etoposide altered the expression of common DEGs, with the exception of *PPM1D* and *TIGAR* (Fig. 7). This suggests that TNBC cells may employ similar signaling pathways to respond to DNA damage stress induced by different chemotherapeutic agents.

### The regulatory role of TP53 in the expression of common upregulated DEGs in response to etoposide

**Fig. 8:**
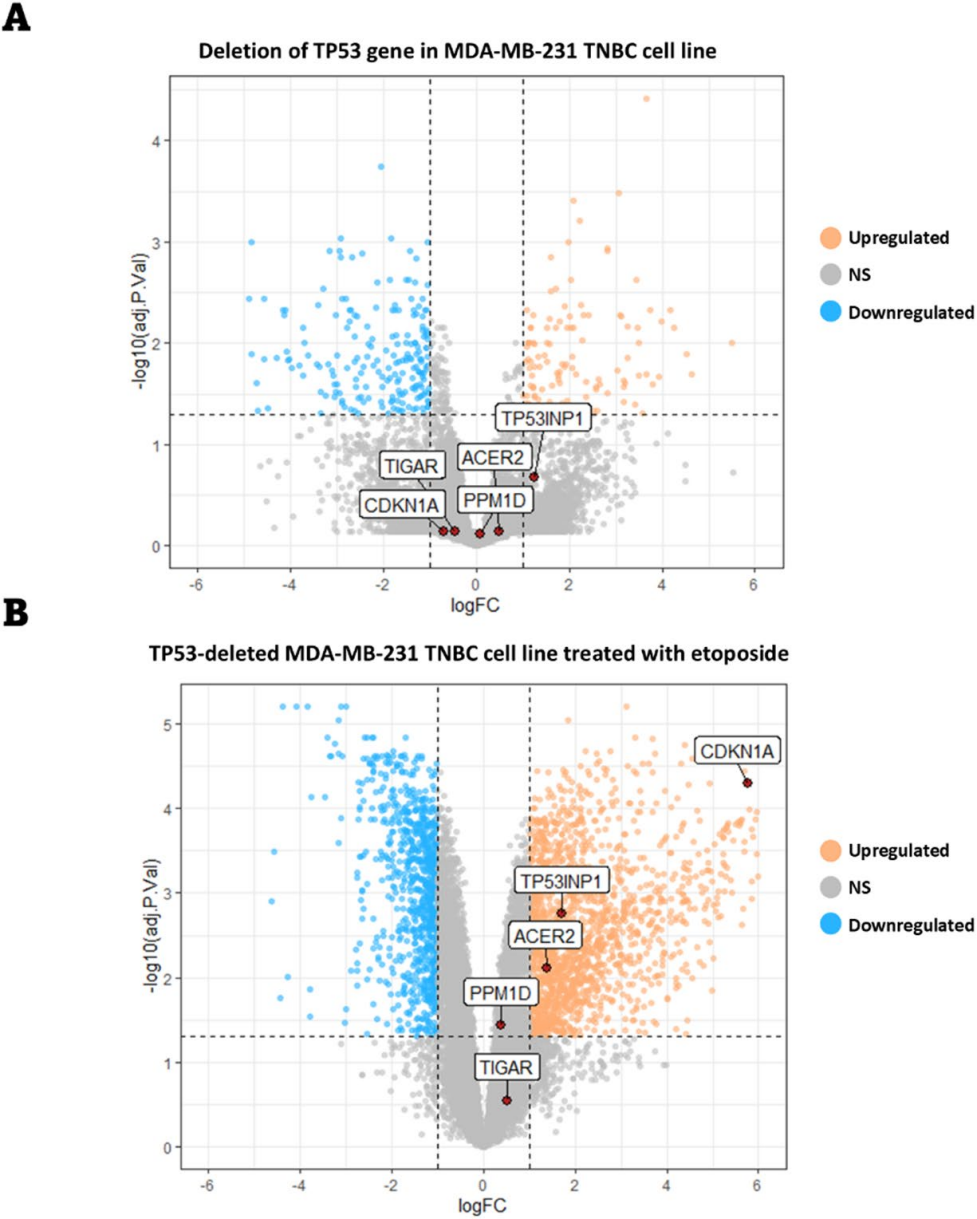
Expression of common DEGs in TP53-Deleted MAD-MB-231 cells. (**A**) Expressions of common DEGs in MDA-MB-231 cells expressing mutated TP53 were compared with T53-deleted cells using RNA-seq data from the GSE181010 dataset. (**B**) The effect of etoposide treatment on the expression of common DEGs in TP53-deleted MAD-MB-231 cells was evaluated. The data are presented as a volcano plot, with the x-axis representing the logFC and the y-axis representing the -log 10 of the adj.P.value. Genes with logFC ≥ 1 and adj.P.value ≤ 0.05 were considered as a DEG.

Our PPI network analysis revealed that TP53 has experimentally validated interactions with TIGAR, PPM1D, CDKN1A, and TP53INP1. To determine whether the expression of these genes is regulated by TP53 following treatment with DNA-damaging drugs, the dataset GSE 181010 were utilized for further analysis^16^. In this dataset, the *TP53* gene, which harbors the R280K mutation in MDA-MB-231 TNBC cells, was deleted using the CRISPR/Cas9 system. The TP53 knock out (KO) cells were then treated with etoposide to investigate the impact of TP53 deletion on cellular response. Analysis of the dataset showed that TP53 deletion alone did not significantly alter the expression of common DEGs (Fig. 8A). However, treatment with etoposide, a DNA-damaging drug, induced the expression of *CDKN1A*, *TP53INP1*, and *ACER2* in TP53-deleted TNBC cells (Fig. 8B). This suggests that the expression of these genes in response to cellular stress can occur independent of TP53 function. Taken together, these findings indicate that TP53 plays a partial role in regulating the response of TNBC cells to DNA-damaging agents. Further studies are warranted to fully elucidate the role of TP53 in the cellular response to DNA damage stress.

### Correlation between drug sensitivity and common DEGs dependency in TNBC cell lines

To evaluate the relationship between cancer cell dependency on common DEGs expression and sensitivity to DNA-damaging drugs, we analyzed TNBC cell lines using the DepMap database. We assessed the correlation between CRISPR-mediated knockout of common DEGs, measured through changes in cell viability, and the sensitivity of these cells to four DNA-damaging agents including doxorubicin, epirubicin, etoposide, and mitoxantrone. In this analysis, negative dependency score indicates that gene deletion reduces cell viability, while a positive score suggests that knockout may enhance cell growth, implying a potential tumor suppressor-like role. Both Pearson and Spearman correlations were calculated, and results with *p* < 0.05 were considered statistically significant (Table 4).

**Fig. 9:**
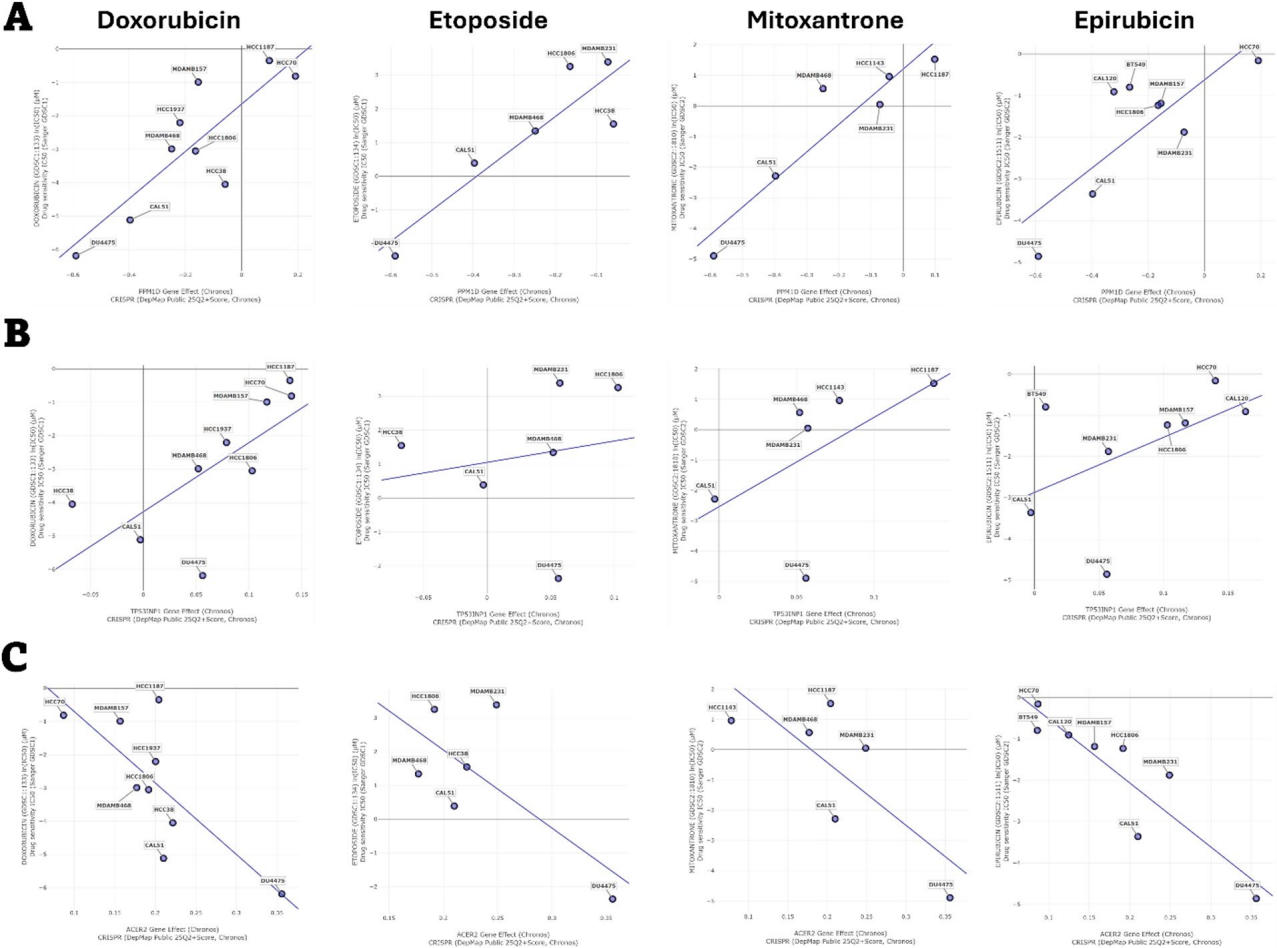
Correlation between drug sensitivity and common DEGs dependency in TNBC cell lines. The Y-axis shows the log-transformed IC50 values (µM) of four DNA-damaging drugs including doxorubicin, epirubicin, etoposide, and mitoxantrone. The X-axis shows gene effect scores obtained from CRISPR-Cas9 loss-of-function screens using the Chronos model. Data are presented for (**A**) *PPM1D*, (**B**) *TP53INP1*, and (**C**) *ACER2* genes. Statistically significant correlations were defined as *p* < 0.05, as reported in Table 2.

Our findings revealed that higher dependency of the TNBC cells on *PPM1D* was significantly correlated with increased sensitivity to all four drugs, underscoring its important role in drug response (Fig. 9A). A similar correlation was observed between dependency on *TP53INP1* and sensitivity to doxorubicin (Fig. 9B). Conversely, deletion of *ACER2* promoted higher cell growth, which was correlated with improved response to doxorubicin and epirubicin (Fig. 9C). This suggests a strong tumor-suppressive function of ACER2 in TNBC and its association with enhanced drug sensitivity. No significant correlations were found between other common DEGs and sensitivity to DNA-damaging drugs. Overall, these results indicate that dependency on common DEGs is closely associated with TNBC cell responses to DNA-damaging therapies, and may serve as potential predictive markers for treatment outcomes or novel target to improve the efficacy of current treatments.

**Table 4:** Correlations between gene dependency scores and drug sensitivity in TNBC cell lines. Correlation values were calculated using both Pearson and Spearman models.

Gene	Drug	Pearson	Spearman	*p*-value
ACER2	Doxorubicin	−0.761	−0.683	0.017
ACER2	Etoposide	−0.704	−0.143	0.118
ACER2	Mitoxantrone	−0.770	−0.771	0.073
ACER2	Epirubicin	−0.903	−0.952	0.0021
CDKN1A	Doxorubicin	−0.100	−0.050	0.79
CDKN1A	Etoposide	0.431	0.257	0.39
CDKN1A	Mitoxantrone	−0.182	−0.486	0.73
CDKN1A	Epirubicin	0.323	0.238	0.43
PPM1D	Doxorubicin	0.840	0.767	0.0046
PPM1D	Etoposide	0.898	0.829	0.014
PPM1D	Mitoxantrone	0.935	0.943	0.0062
PPM1D	Epirubicin	0.791	0.571	0.019
TIGAR	Doxorubicin	−0.315	−0.250	0.40
TIGAR	Etoposide	−0.064	−0.257	0.90
TIGAR	Mitoxantrone	0.172	0.143	0.74
TIGAR	Epirubicin	−0.230	0.190	0.58
TP53INP1	Doxorubicin	0.710	0.800	0.032
TP53INP1	Etoposide	0.176	0.429	0.73
TP53INP1	Mitoxantrone	0.550	0.714	0.25
TP53INP1	Epirubicin	0.519	0.548	0.18

## Discussion

DNA-damaging drugs are crucial in treating various types of cancers, including BC. To date, several drugs have been developed to target the genome of cancer cells and induce cellular stress. Among these, doxorubicin, epirubicin, and etoposide are prominent, with doxorubicin being the most widely used for treatment of various types of cancers.

Various mechanisms of action and resistance have been proposed for doxorubicin, as comprehensively reviewed by Micallef et al. and Mattioli et al.^11,47^. Doxorubicin intercalates between DNA strands, and trap topoisomerase II which leads to an increase in DNA DSBs, inhibition of DNA replication, and ultimately cell cycle arrest and apoptosis in cancer cells^6,7,48,49^. Another key mechanism involves the generation of reactive oxygen species (ROS) within cells, which damages critical cellular components, including DNA^50–53^.

Although doxorubicin has emerged as a promising anti-cancer drug, some caners develop resistance to it through the activation of various signaling pathways. Research has demonstrated that the phosphoinositide 3 kinase (PI3K) and Mitogen-Activated Protein Kinase (MAPK) signaling pathways play an important role in mediating resistance to doxorubicin in cancer cells^54–57^. Moreover, cancer cells with efficient DNA repair mechanisms can resist doxorubicin^58,59^.

Since the approval of doxorubicin in 1974 for treating metastatic BC, numerous clinical trials have demonstrated its efficacy in improving patient survival rates^13,60^. Despite the proven effectiveness of doxorubicin, both as a standalone treatment and in combination with other anticancer drugs, some TNBC patients develop resistance to it. The limited understanding of doxorubicin’s molecular mechanisms of action in different types of cancer, as well as the mechanisms underlying drug resistance, poses significant challenges to enhance its efficacy or overcome resistance.

In this study, we aimed to investigate the initial response of doxorubicin-sensitive TNBC cells to the drug. We then examined the expression patterns of activated genes in various types of BC and in doxorubicin-resistant TNBC cells. Next, we extended our exploration to observe the same phenomenon in TNBC cells treated with other drugs that share similar mechanisms of action. Finally, we investigated whether the dependency of TNBC cells on upregulated genes correlates with their sensitivity to DNA-damaging drugs.

In this study, we utilized three TNBC cell lines, HCC1806, CAL-51, and MAD-MB-231, to investigate the mechanism of action of doxorubicin. According to the TP53 Database^44^, the *TP53* status of these cell lines, which influences their response to DNA damage, varies. CAL51 possesses a WT *TP53* gene, whereas HCC1806 and MDA-MB-231 harbor loss of function mutations. MDA-MB-231 cells carry the R280K mutation in the *TP53* gene, located within the DNA binding domain^16,61^. The arginine at position 280 is critical for p53’s ability to bind DNA^61^, and mutations in this region impair p53’s DNA-binding capacity^62^. In addition, the HCC1806 cell line harbors a *TP53* frameshift mutation, an alteration that typically results in complete loss of normal p53 activity^63^.

We assessed the sensitivity of TNBC cell lines to various DNA-damaging drugs using the GDSC databases. Our analysis revealed that the CAL-51 and MDA-MB-231 cell lines were more sensitive to these drugs compared to other TNBC cell lines.

To explore the effects of doxorubicin on gene expression patterns in TNBC cell, we analyzed the GSE174692 and GSE202536 datasets. Our findings indicated that five genes (*CDKN1A*, *TIGAR*, *TP53INP1*, *PPM1D*, and *ACER2*) were upregulated, while two genes (*ASNS*, *TRIB3*) were downregulated in both datasets. Interestingly, our PPI network analysis revealed that all upregulated genes except ACER2 interact with p53 protein. A review of the literature further confirmed that all upregulated genes, including *CDKN1A*^64,65^, *TP53INP1*^66,67^, *TIGAR*^68,69^, *PPM1D*^70^, and *ACER2*^45^ are direct targets of p53. Functional enrichment analysis further demonstrated that these genes are involved in cellular response to stress, radiation, starvation, and DNA damage suggesting that specific signalling pathway are activated in TNBC cells in response to the cellular stress induced by doxorubicin.

The upregulated genes *CDKN1A*, *TP53INP1*, *TIGAR*, and *ACER2*, have all been previously described as having either oncogenic or tumor-suppressive roles, depending on the cell context. Therefore, this duality suggests two potential scenarios for the function of this activated signaling pathway. Furthermore, the expression of these genes can be regulated through either p53-dependent or p53-independent mechanism, as evidenced by the fact that CAL-51 has WT p53 while HCC1806 does not express functional p53. As an example of p53-independent pathway, *TP53INP1* expression can be activated independently of p53 by transcription factors like p73^71^ and E2F1^72^. Supporting this, we showed that etoposide which acts similar to doxorubicin, upregulates *CDKN1A*, *TP53INP1*, and *ACER2* independently of p53 function. This suggests that the activation of this pathway is partially influenced by both the TNBC cell type and the specific DNA-damaging drug. Overall, further studies are needed to comprehensively investigate the regulation of these genes in the presence and absence of p53, and to define their potential role in cellular responses to DNA damage.

In the first scenario, doxorubicin treatment increases intracellular ROS and causes extensive DNA damage in TNBC cells. The upregulation of common DEGs promotes cell survival by facilitating DNA repair and reducing ROS levels. When DNA damage occurs during the G1 phase, increased expression of *CDKN1A* can redirect the cell back to G0 phase^73^. In addition, DNA damage during the S phase triggers CDKN1A-mediated cell cycle arrest in postmitotic cells at the G1 phase (G1pm)^74^. The outcome of this cell cycle arrest can be promoting the DNA repair and continued cell cycle progression which depends on the expression level of *CDKN1A* and the cell’s ability to overcome the DNA damage stress^75,76^. Indeed, there is direct evidence suggesting that CDKN1A may reduce the sensitivity of cancer cells to doxorubicin-induced cell death. For instance, Ma et al. demonstrated that increased *CDKN1A* expression, mediated by p65 (p53-independent pathway) in colorectal carcinoma cells, contributes to resistance to doxorubicin-induced apoptosis^77^. Furthermore, it has been demonstrated that the binding of CDKN1A to pro-caspase-3 inhibits caspase-3 activation, thereby suppressing doxorubicin-induced cell death^78^. Therefore, CDKN1A can play a critical role in the cellular response to DNA damage potentially by arresting the cell cycle and inhibiting apoptosis through suppression of caspase activation. Interestingly, doxorubicin-resistant CAL51 cells not only show elevated baseline expression of *CDKN1A* compared to sensitive cells but also further induce its expression upon doxorubicin treatment which may support its protective role against DNA damages. In addition, TP53INP1 can potentially act as pro-survival protein helping the cell to overcome the toxic effects of doxorubicin. Indeed, it has been suggested that cytoplasmic TP53INP1 positively regulates autophagy, which could potentially promote cancer cell survival through the elimination of damaged organelles^79,80^. The protective role of TP53INP1 is supported by evidence showing that its silencing sensitizes human fibroblast cells to radiation^81^. Consistent with this, our results demonstrate that *TP53INP1* is upregulated in doxorubicin-resistant CAL51 cells. This elevated expression may help resistant cells activate autophagy to clear organelles damaged by doxorubicin-induced ROS. A similar context-dependent role may exist for ACER2. ACER2 is an enzyme that hydrolyzes ceramide into sphingosine^82^. Although higher expression of *ACER2* induces apoptosis, its lower upregulation could promote cell survival^83^, indicating that expression level dictates functional outcome. This potential oncogenic role is corroborated by other studies. Zhang et al. reported that TIMELESS circadian regulator (TIM) promotes BC proliferation via ACER2 and that ectopic *ACER2* expression rescues growth in TIM-deficient cells, affirming its tumor-promoting capacity^84^. Similarly, ACER2 enhances growth and metastasis in hepatocellular carcinoma (HCC)^85^, underscoring its function as an oncogene in certain contexts. Finally, in this proposed scenario, TIGAR upregulation could enable cell survival by mitigating the ROS stress induced by doxorubicin. This is mechanistically supported by studies demonstrating that TIGAR activates the pentose phosphate pathway to reduce intracellular ROS levels and counteract ROS-mediated damage^86,87^. The oncogenic relevance of TIGAR is further highlighted by in vivo evidence showing that its overexpression in MDA-MB-231, T47D, and MCF7 BC cells promotes tumor growth in a xenograft mouse model^88^. Our analysis also indicates that TIGAR expression in TNBC tumor samples, though not statistically significant, is elevated compared to healthy controls.

The second scenario, which we consider more likely, is that doxorubicin induces intracellular ROS and DNA damage, activating the CDKN1A/TP53INP1/TIGAR/ACER2 axis. This activation triggers cell cycle arrest and leads to cell death via either p53-dependent or -independent mechanisms. A key finding supporting the tumor-suppressive role of this pathway is its significant downregulation in doxorubicin-resistant cells.

CDKN1A, also known as p21, regulates cell cycle progression by directly interacting with cyclin-dependent kinases (CDKs) and cyclins, thereby inhibiting their function and arresting cell cycle^89,90^. This cell cycle arrest can be continued by inducing apoptosis in cancer cells via other upregulated genes. Indeed, numerous studies have highlighted the tumor-suppressive role of CDKN1A in various types of cancers^75^. For example, research has shown that the promoter region of the *CDKN1A* gene is significantly more methylated in BC patients compared to healthy individuals^91^. In addition, analysis of BC patient samples have revealed reduced expression of *CDKN1A* in tumor tissues compared to healthy controls^75^. In our analysis of BC patient samples, we also observed that *CDKN1A* is downregulated in TNBC, although this finding was not statistically significant. In addition, *TP53INP1* expression is also upregulated in response to various cellular stresses, such as oxidative stress and DNA damage to promote cell cycle arrest and apoptosis^80^. Moreover, *TP53INP1* expression is significantly reduced in several types of cancers, including colorectal cancer, pancreatic adenocarcinoma, and melanoma^80^. In breast carcinoma, *TP53INP1* expression is decreased in approximately 56% of cases^92^. Although, our analysis revealed that the *TP53INP1* gene is amplified in about 23% of BC cell lines and 16% of tumor samples, our further analysis confirmed that this increase mostly occurs in luminal A and B subtypes of breast tumors compared to normal breast tissue. Interestingly, comparing *TP53INP1* expression levels between TNBC tumors and other BC subtypes demonstrated the lower expression of this gene in TNBC tumors, suggesting that the role of *TP53INP1* may differ between TNBC and other BC subtypes. Additionally, unlike other subtypes of breast tumors, *TP53INP1* expression in TNBC tumors, though not statistically significant, tends to be lower compared to healthy controls which may support its tumor-suppressive role in TNBC cells. Mechanistically, TP53INP1 enhances p53 function through a positive feedback loop; it inhibits p53’s degradation by promoting its phosphorylation at serine 46^93,94^. Moreover, as discussed before cytoplasmic TP53INP1 activate autophagy which leads to cell death based on the findings of these studies^79,80^. Beyond CDKN1A and TP53INP1, ACER2 also plays a critical role in the DNA damage response and multiple lines of evidence support its role as a tumor suppressor in cancer cells. For instance, Xu et al. demonstrated that DNA damage induced by doxorubicin increases *ACER2* expression, leading to sphingosine generation, sphingosine-mediated ROS production, and ultimately cell death^95,96^. Our analysis indicates that *ACER2* expression is downregulated in TNBC tumor compared to healthy controls, suggesting its potential tumor-suppressive role. This is supported by the deletion of the *ACER2* gene in approximately 10% of BC cell lines. Furthermore, data from the DepMap database reveals that knocking out *ACER2* increases cell growth in some TNBC cell lines, providing functional evidence for its tumor-suppressive function. In addition, we found that a stronger tumor-suppressive signature is correlated with greater sensitivity to doxorubicin and epirubicin, linking ACER2’s role to drug response. Finally, TIGAR can play a crucial role in reprograming cellular metabolism by inhibiting glycolysis and activating the pentose phosphate pathway^68^. Its tumor-suppressive function may stem from this metabolic shift; by restricting glycolytic energy production, TIGAR can inhibit cancer cell proliferation^97^. Supporting this, Madan et al. demonstrated that *TIGAR* knockdown promotes a pro-proliferative gene expression signature and suppresses cell cycle arrest in tamoxifen-treated MCF7 BC cells^98^.

PPM1D regulates key cellular processes, including the cell cycle and DNA damage repair and functions as a major inhibitor of the p53^99^. It is amplified in approximately 11% of breast tumors, and its overexpression is associated with poor clinical outcomes^100,101^. Mechanistically, PPM1D dephosphorylates p53 at serine-15, inhibiting its activity and promoting cell cycle progression^70,101^. This creates a negative feedback loop, where cellular stress induces p53, which then activates *PPM1D* expression to dampen the p53 response. Moreover, several studies have confirmed *PPM1D* amplification in breast tumors, noting that its overexpression primarily occurs in tumors with WT *TP53*^102,103^. Our results align with this, showing *PPM1D* amplification in about 8% of breast tumors, although this amplification is not correlated with increased gene expression across different BC subtypes.

The oncogenic role of PPM1D is well-established, particularly in a p53-dependent context. Kong et al. demonstrated that inhibiting *PPM1D* expression sensitizes MCF7 BC cells to doxorubicin by increasing p53 phosphorylation and the expression of pro-apoptotic genes^104^. Similar findings in colon cancer confirm that *PPM1D* knockdown increases sensitivity to chemotherapeutics^105^. This evidence strongly supports the second scenario we proposed, where PPM1D acts as an oncogene by blunting the DNA damage response.

It is critical to emphasize that PPM1D’s oncogenic function depends on the presence of WT p53 and its role in p53-mutant cells requires further investigation. This distinction aligns with the fundamental functional difference between WT and mutant p53, where the loss of WT p53 can promote cell survival, while the loss of certain p53 mutants may have no effect^16^. Interestingly, our data revealed that higher cellular dependency on the *PPM1D* gene is correlated with increased sensitivity to DNA-damaging drugs in TNBC cells. This suggests that TNBC cells relying on PPM1D for survival are more vulnerable when treated with these drugs, positioning PPM1D as a critical determinant of therapeutic response.

## Conclusion

Our findings demonstrate that one of the important phenomena when treating TNBC cells with DNA-damaging drugs, such as doxorubicin and etoposide, is the activation of TP53 target genes expression, a process that is significantly impaired in drug-resistant cells. However, the regulation of these genes requires to further investigation, as our results, along with those of others, suggest that these genes can also be regulated through TP53-independent pathways. We propose that doxorubicin treatment activates a signaling pathway in TNBC cells that can lead to one of two potential outcomes. In a pro-survival response, the pathway facilitates repair: CDKN1A arrests the cell cycle, TP53INP1 and TIGAR promote autophagy and reduce ROS to mitigate damage, and ACER2 generates pro-survival sphingosine. In a tumor-suppressive response, the same genes drive cell death: CDKN1A arrest cell cycle, TP53INP1 induces lethal autophagy and stabilizes p53, TIGAR inhibits glycolysis to reduce energy generation, and ACER2 produces pro-apoptotic sphingosine. In both scenarios, PPM1D provides negative feedback on p53. Based on our data and prior studies, the tumor-suppressive outcome is more probable; however, further research is needed to define the regulatory mechanisms that determine this pathway’s role in the efficacy of DNA-damaging drugs.

## Data Availability

The datasets analysed during the current study are available in the Gene Expression Omnibus (GEO) repository under the accession numbers GSE174692 [https://www.ncbi.nlm.nih.gov/geo/query/acc.cgi? acc=GSE174692], GSE181010 [https://www.ncbi.nlm.nih.gov/geo/query/acc.cgi? acc=GSE181016], and GSE202536 [https://www.ncbi.nlm.nih.gov/geo/query/acc.cgi? acc=GSE202536].
